# Drug–Drug Interactions Involving Intestinal and Hepatic CYP1A Enzymes

**DOI:** 10.3390/pharmaceutics12121201

**Published:** 2020-12-11

**Authors:** Florian Klomp, Christoph Wenzel, Marek Drozdzik, Stefan Oswald

**Affiliations:** 1 Institute of Pharmacology and Toxicology, Rostock University Medical Center, 18057 Rostock, Germany; florian.klomp@uni-rostock.de; 2 Department of Pharmacology, Center of Drug Absorption and Transport, University Medicine Greifswald, 17487 Greifswald, Germany; Christoph.Wenzel@med.uni-greifswald.de; 3 Department of Experimental and Clinical Pharmacology, Pomeranian Medical University, 70-111 Szczecin, Poland; marek.drozdzik@pum.edu.pl

**Keywords:** cytochrome P450, CYP1A1, CYP1A2, drug–drug interaction, expression, metabolism, regulation

## Abstract

Cytochrome P450 (CYP) 1A enzymes are considerably expressed in the human intestine and liver and involved in the biotransformation of about 10% of marketed drugs. Despite this doubtless clinical relevance, CYP1A1 and CYP1A2 are still somewhat underestimated in terms of unwanted side effects and drug–drug interactions of their respective substrates. In contrast to this, many frequently prescribed drugs that are subjected to extensive CYP1A-mediated metabolism show a narrow therapeutic index and serious adverse drug reactions. Consequently, those drugs are vulnerable to any kind of inhibition or induction in the expression and function of CYP1A. However, available in vitro data are not necessarily predictive for the occurrence of clinically relevant drug–drug interactions. Thus, this review aims to provide an up-to-date summary on the expression, regulation, function, and drug–drug interactions of CYP1A enzymes in humans.

## 1. Introduction

The oral bioavailability of many drugs is determined by first-pass metabolism taking place in human gut and liver. In this regard, a considerable fraction of the administered dose is presystemically eliminated by intestinal and hepatic phase I and/or phase II drug metabolism Consequently, only a minor fraction of the administered dose reaches the central compartment and in turn the site of action. Thus, alterations of the aforementioned presystemic metabolism in terms of inhibition or induction of the involved metabolizing enzymes may result in two unwanted clinical scenarios: (1) increased drug exposure as caused by enzyme inhibition with an increased risk of side effects up to drug-related toxicity, and (2) subtherapeutic drug levels due to induction of the respective metabolizing enzymes, which may threaten the therapeutic drug effects [1,2].

During the last decades, it was clearly demonstrated that major cytochrome P450 (CYP) enzymes such as CYP3A4, CYP2C9/19, and CYP2D6 play a major role in first-pass metabolism of drugs [3,4,5]. Here, extensive pharmacokinetic and pharmacogenetic studies have been conducted and identified these enzymes as crucial determinants in the pharmacokinetics and, in turn, for efficacy and safety of their substrates [6,7,8,9]. However, beside these major enzymes, the information about other CYPs that are considerably expressed in the human intestine or liver and significantly involved in the metabolism of frequently used drugs is much more limited. Examples for these somewhat “under-investigated” enzymes are CYP1A1 and CYP1A2, which are involved in the metabolism of about 10% of the drugs on the market [10,11]. Despite their clinical relevance, considerably fewer studies related to human pharmacokinetics and drug–drug interactions compared to the above-mentioned major enzymes have so far been published. For example, Medline search (via PubMed^®^) on “human pharmacokinetics” and certain enzymes listed 6379 entries for CYP3A4, 2902 for CYP2C9/19, and 2794 for CYP2D6, but “only” 734 and 1749 have been found for CYP1A1 and CYP1A2, respectively (assessed 22 October, 2020). Thus, the aim of this mini review article is to provide an up-to-date overview about the current knowledge on the expression, regulation, and clinically relevant drug–drug interactions of CYP1A1 and CYP1A2 in humans.

## 2. Expression

CYP1A1 and CYP1A2 belong to the CYP1 gene family; they are highly conserved and located on chromosome 15 [10,12]. CYP1A2 is constitutively expressed at high levels in human liver, whereas CYP1A1 was shown to be expressed at markedly lower levels in the organ but is also found in extrahepatic tissues including lung, intestine, prostate, kidney and placenta [11,13,14,15,16,17,18,19]. However, data on the protein abundance of hepatic CYP1A1 are conflicting; some studies demonstrated its absence in human liver, while others could clearly quantify the enzymatic protein [14,16,18,20,21,22,23]. In particular, recent mass spectrometry-based studies have demonstrated a substantial abundance of CYP1A1 in the human liver ranging from 0.5 to 9 pmol/mg microsomal protein [18,23].

In general, the available data on CYP1A1/2 expression are somewhat limited. There are sporadic studies on intestinal or hepatic CYP1A1 and/or CYP1A2 gene expression or protein abundance that are partly inconsistent and conflicting (Table 1). To overcome this limitation counteracting reliable conclusions, especially on the role of intestinal and hepatic CYP1A1, comprehensive information about gene and protein expression of CYP1A1 and CYP1A2 in intestinal and hepatic tissues from the same individuals are needed. So far, only one study from our group is available that included intestinal and hepatic tissue samples in parallel from organ donors, in order to overcome the known issue of high inter-subject variability in the expression of metabolizing enzymes [24]. However, this study only considered CYP1A2, but not CYP1A1.

**Figure 1:**
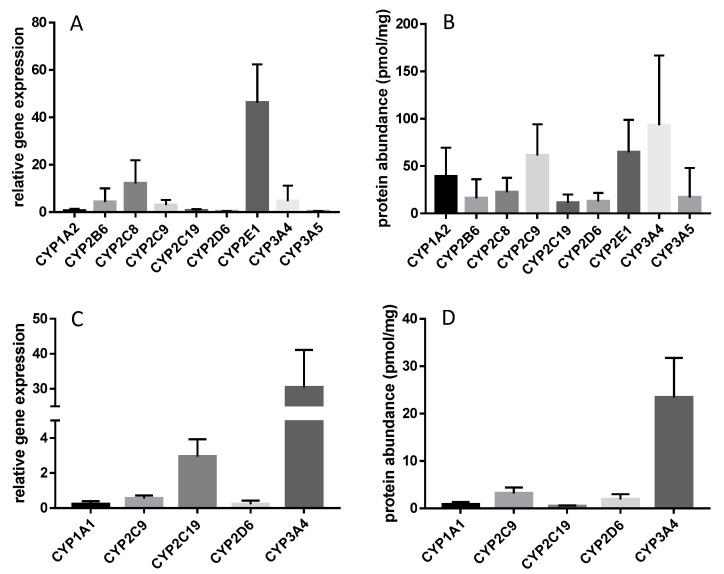
Comparative intestinal and hepatic gene expression and protein abundance of CYP1A1 and CYP1A2 (mean ± SD). Relative gene expression and absolute protein abundance of clinically relevant CYP450 enzymes in the human liver is presented in section (**A**,**B**) (data taken from Drozdzik et al. 2019 (**A**) and Achour et al. 2014 (**B**)). Sections C and D show relative gene expression and absolute protein abundance of clinically relevant CYP450 enzymes in the human small intestine observed in 30 ((**C**), own unpublished data) and 28 human jejunal tissue samples ((**D**), Miyauchi et al. 2016).

Compared to CYP3A4 and CYP2C9, which are the most abundant intestinal CYP enzymes [16,24], CYP1A1 expression is rather little in the intestine and highly variable as well [16,30] (Figure 1). Paine et al. were able to detect CYP1A1 enzyme in only three of 31 investigated human jejunal samples at a range of 3.6 to 7.7 pmol/mg (Western blotting), while Miyauchi et al. using the targeted proteomics approach found CYP1A1 in 15 out of 28 analyzed human small intestinal samples (range: 0.07–2.3 pmol/mg) [16,30]. In parallel, also Shrivas et al. found CYP1A1 only in three out of 32 human liver microsomal samples using global proteomics [23]. Those findings indicate a substantial inter-subject variability in CYP1A1 protein that is most likely attributed to the individual lifestyle, including exposure to different diets or smoking, which were already shown to be important determinants of variability in the expression as well as the metabolic function of CYP1A enzymes [13,21,27,33]. In addition, experimental conditions may have partly contributed to the observed variability.

In contrast to the mentioned controversies on CYP1A1, CYP1A2 is doubtlessly expressed at considerable levels in the human liver, but not in the intestine (Table 1). A meta-analysis on the expression of 15 hepatic drug-metabolizing enzymes revealed that CYP1A2 contributes about 10% to all CYP enzymes [34]. After CYP3A4, CYP2E1, and CYP2C9, CYP1A2 showed the fourth highest protein abundance of all investigated hepatic CYP enzymes. These data have been confirmed by more recent mass spectrometry-based studies [24,31,35,36,37]. The mean hepatic microsomal protein abundance ranged from 14 to 35 pmol/mg, as determined by targeted proteomics [18,22,28,31,36,38]. Former data as derived from immunostaining studies determined considerably higher abundance, up to 65 pmol/mg [39,40], which might be interpreted with caution considering the issue of unspecific binding of antibodies. As a general feature of CYP1A1 and CYP1A2 expression, one must consider a substantial inter-subject variability, which seems to be caused by a complex interplay of genetic, epigenetics, and environmental factors [11,41].

## 3. Regulation

### 3.1. Transcriptional Regulation

The promoter region of CYP1A1 and CYP1A2 genes contains several Aryl hydrocarbon receptor (AhR) response elements [42,43], which, after binding of respective compounds, initiate coordinated transcription of both genes. Consequently, both genes are highly inducible by AhR ligands such as polycyclic aromatic hydrocarbons (PAHs), dioxins and numerous xenobiotics [44]. Examples for well-established experimental inducers are 2,3,7,8-tetrachlorodibenzo-p-dioxon (TCDD), 3-methlycholanthrene and β-naphthoflavone [45,46]. In addition, omeprazole was shown to be a potent model inducer for CYP1A1 and CYP1A2 [47,48,49]. There is evidence that the induction of CYP1A1 by AhR is stronger than that of CYP1A2 [44,49,50]. This is also in agreement with the fact that tissues possessing high expression of CYP1A1 (i.e., lung, placenta, intestine, urinary bladder) also show high expression levels of AhR [51]. Table 2 provides an overview of in vitro, in vivo, and ex vivo data on CYP1A1/1A2 induction by clinically relevant drugs. In this regard, albendazole, carbamazepine, omeprazole, lansoprazole, primaquine, and rosiglitazone were shown to be strong inducers of both, expression and metabolic activity of CYP1A1 and CYP1A2 [48,49,50,52]. As discussed later, these findings do not necessarily translate for all compounds to clinically relevant drug–drug interactions (e.g., for omeprazole). Relevant exogenous sources of AhR activators are charcoal grilled food, tobacco smoking as well as other natural sources including broccoli or fish oil supplementation, which strikingly induce endogenous eicosanoids [11,53,54]. Consequently, some of these environmental factors may contribute to the substantial variability in the expression and function of both CYP1A isoenzymes. Associated with this—tobacco smoking, especially, was shown to have significant effects on the pharmacokinetics and actions of many CYP1A substrates (see paragraph “Drug–Drug Interactions”).

As already described for genes regulated by other nuclear receptors (e.g., *ABCB1* by pregnane-X-receptor (PXR)), a partial transactivation of human CYP1A by nuclear receptors other than AhR is possible. In this regard, CYP1A1 and 1A2 were also shown to be induced upon activation of the human constitutive androstane receptor (CAR) [63]. This explains considerable induction of CYP1A enzymes by typical CAR ligands, such as carbamazepine, phenobarbital and phenytoin. On the other side, the relevance of PXR in the regulation of CYP1A seems to be negligible as shown in vitro [47,59,60] and in vivo [64].

### 3.2. Impact of Gender, Age, and Diseases

In addition to the described transcriptional regulation, also several nongenetic factors seem to influence CYP1A2 expression and function. For example, protein abundance and metabolic CYP1A2 activity for different substrates was shown to be considerably lower in woman than in men [31,65,66,67]. However, as smoking and the intake of oral contraceptives (inhibitors of CYP1A function) represent substantial confounders of CYP1A2 expression and function, those data have to be interpreted with caution and need further verification. Moreover, in analyzing potential gender differences in the pharmacokinetics of CYP1A substrates, dose-adjustment was shown to be essential as demonstrated for tizanidine [68]. There is also evidence that CYP1A2 activity is significantly higher in younger (<20 years) than in older people (>20–60 years and >60 years) [65].

Disease-related changes have been also reported for CYP1A. Here, CYP1A2 expression in liver dysfunction and cholestasis was found to be decreased [40,69]. Other studies failed to confirm those differences in vivo [53,54] and in human liver tissue at both, mRNA and protein levels [40,70]. More recent mRNA expression data demonstrated that the expression of CYP1A2 was decreased by about 90% in hepatocellular carcinoma livers, 80% in alcoholic cirrhosis, and 65% in severe cirrhosis [71]. In parallel, analysis of liver biopsy samples of patients with chronic hepatitis C revealed significantly lower gene expression levels of CYP1A1 and CYP1A2 [72,73]. These data have been recently confirmed by a targeted proteomic analysis [74]. Likewise, nonalcoholic fatty liver disease (NAFLD) was associated with decreased mRNA, protein amount, and functional activity of microsomal CYP1A2 compared to healthy liver tissue [75].

### 3.3. Genetics and Epigenetics

The large inter-individual variability in the elimination of drugs undergoing CYP1A2 metabolism has been attributed to genetic and environmental factors [11,76,77]. In this regard, Rasmussen and colleagues demonstrated in a large study in 378 mono- and dizygotic twins for the caffeine metabolic ratio (a surrogate for CYP1A2 activity) a strong overall heritability of 0.72 [78].

Common polymorphisms in the CYP1 gene have been found to be only of limited relevance for human drug metabolism. However, considering the involvement of CYP1A enzymes in bioactivation of procarcinogens, many studies investigated certain single nucleotide polymorphisms in association to various types of cancer [10,11]. The Pharmacogene Variation Consortium website (www.pharmvar.org) lists 15 alleles for CYP1A1 [79]. Of the most frequent variants m1 to m4, only the common non-synonymous variant CYP1A1*2C (rs1048943, 2454A>G, Ile462Val), which has a global minor allele frequency of about 12%, was shown to be associated with substantially modified enzymatic activity, i.e., 6- to 12-fold higher for its substrates 17β-estradiol and estrone [80]. This variant was associated with an increased risk for lung cancer in Chinese and breast, and prostate cancer in Caucasians [10,11].

Several alleles, namely 24, have been also reported for CYP1A2 [79], of which only the most established will be briefly mentioned here. The CYP1A2*6 variant was shown to result in a nonfunctional protein [81]. However, considering the rare occurrence of this and other variants [82], they are expected to be of limited clinical relevance. The CYP1A2*1C was associated with reduced CYP1A2 induction by cigarette smoking in Japanese [83]. On the contrary, the CYP1A2*1F variant (–163C>A) was linked with enhanced enzyme inducibility in Caucasian smokers [53,84] and heavy coffee drinkers [85]. Interestingly, carriers of the combined genotype CYP1A2*1C/*1F were not inducible by the AhR ligand omeprazole [86]. Both variants were described to increase the susceptibility to certain cancers. Despite the described multiplicity of CYP1A2 polymorphisms, clear gene dose relationships by comparing common SNPs to the respective protein abundance or metabolic phenotype could not be demonstrated yet. Thus, so far no single SNP or haplotype in the CYP1A2 gene seems to be predictive [41]. In this regard, a multivariate linear modeling by Klein et al. revealed that genetic polymorphisms contribute about 35% of hepatic CYP1A2 activity variation, whereas some 40% of the variation were explained by nongenetic factors together [40].

However, the clinical impact of genetic variation in terms of susceptibility factors for cancer or pharmacokinetics, efficacy and safety of certain CYP1A substrates is not systematically covered here but was excellently summarized by others [10,11,87,88,89,90].

Finally, there is also evidence for an epigenetic regulation of CYP1A2 expression as concluded from the observation that the extent of DNA methylation of a CpG island close to the translation start site was inversely correlated to the hepatic CYP1A2 mRNA expression [53,54]. Recent studies point also to an involvement of certain microRNAs in the expression and induction of CYP1A2 [91,92].

## 4. Metabolic Function, Substrates, and Inhibitors

### 4.1. Metabolic Features

Considering that CYP1A2 shares about 80% amino acid sequence identity with CYP1A1, it is not surprising that the substrate specificities of these enzymes often overlap, owing to a CYP1 family–specific distortion of the F helix in the area of the substrate binding cavity, which produces bending of the helix and results in the formation of an enclosed and planar substrate binding site observed in both CYP1A1 and CYP1A2 [93]. It has been demonstrated that commonly used probe drugs for CYP1A2 such as caffeine, theophylline, phenacetin, propranolol, and 7-ethoxyresorufin are metabolized by both CYP1A isoenzymes [94,95]. Despite this considerable similarity, CYP1A1 shows a preference for planar aromatic hydrocarbons (e.g., naphthalene, PAHs), while CYP1A2 prefers aromatic amines and heterocyclic compounds (e.g., 2-naphthylamine, xanthines) (Table 3). The metabolic feature of CYP1A1 in combination with its expression pattern in tissues potentially exposed to high amounts of PAHs (e.g., the lung via tobacco smoke, the intestine via charbroiled food) makes it plausible that increased expression and function of CYP1A1 may result in higher formation rates of potentially carcinogenic metabolites. In this regard, benzo[a]pyrene and other procarcinogens (e.g., arylarenes, nitroarenes, arylamines) are bioactivated by CYP1A1 to reactive and carcinogenic intermediates such as epoxides which may cause DNA damage and in long term malignancies. In the same manner, CYP1A2 is involved in the bioactivation of heterocyclic aromatic amines (HAAs) originating from cook muscle meats such as beef, pork, or fish to carcinogenic hydroxylamines. Thus, it can be assumed that induction of CYP1A1/1A2 in smokers by inhaling frequently high amounts of PAHs may contribute to strikingly increased risk for lung cancer [96]. However, the toxicological impact of both isoenzymes on the bioactivation of carinogenes from environmental compounds is beyond the scope of this article but summarized elsewhere [11,97].

### 4.2. Substrates

Under the consideration that CYP1A1 is markedly lower expressed in the human liver than CYP1A2 and is also considered to be of extrahepatic relevance, its impact on the metabolic clearance of drugs was formerly assumed to be negligible [10,11]. In contrast to this conclusion, recent studies have clearly verified CYP1A1 protein abundance in human intestine and liver, which challenges the former paradigm of the pharmacokinetically irrelevant CYP1A1 [18,23,30]. Moreover, 15–20 years ago, several studies convincingly demonstrated high metabolic CYP1A1 activity of intestinal and hepatic microsomal fractions [14,27,122]. Associated to this, riociguat (guanylate cyclase stimulator used for the treatment of pulmonary hypertension) and granisetron (5-HT3 receptor antagonist for the treatment of nausea and vomiting following chemotherapy or radiotherapy) were shown to be highly and specifically biotransformed by CYP1A1 [18,122]. In addition, the tyrosine kinase inhibitors axitinib, erlotinib, gefitinib, and ningetinib as well as the toll-like receptor agonist imiquimod and conivaptan (inhibitor of the antidiuretic hormone) have been reported as substrates of CYP1A1 [18,123,124]. Thus, one has to conclude that CYP1A1 should be considered as an additional potentially relevant clearance pathway for some drugs. However, in the past, the metabolic stability of a drug was in most cases studied by using human liver microsomes or recombinant CYP1A2, but not for both isoforms of CYP1A, as done in very recent studies [18,123]. Consequently, the individual contribution of CYP1A1 to the metabolism of established CYP1A2 substrates as summarized in Table 3 remains uncertain, and asks for additional research efforts. However, even today, these kind of head-to-head comparisons of CYP1A1 and CYP1A2 in drug metabolism are challenging because established manufactures of life science consumables (e.g., Thermo Fisher Scientific and Corning) do not provide microsomal preparations of recombinant CYP1A1, but almost exclusively CYP1A2.

It was estimated by analyzing the metabolic pathways of about 250 frequently used drugs, that CYP1A2 is involved in the biotransformation of about 10% of drugs on the market [10]. CYP1A2-typical biotransformation reactions include N-demethylation of caffeine to 1,7-dimethylxanthine (paraxanthine), N-demethylation of clozapine, O-deethylation of phenacetin, and N-demethylation as well as 8-hydroxylation of theophylline. In particular, caffeine and phenacetin were frequently used as probe compounds in vitro and for phenotype determination in vivo [11,125]. Due to its high abundance in the human liver, CYP1A2 plays an important role in the metabolism of many clinically important drugs, including antipsychotics (clozapine, olanzapine), antidepressants (duloxetine, agomelatine, mirtazapine), cardiovascular drugs (propranolol, verapamil), non-steroidal anti-inflammatory drugs (NSAID) (phenacetin), the Alzheimer’s disease drug tacrine, a cholinesterase inhibitor, the muscle relaxant tizanidine, antiparkinson drugs (rasagilin, ropinirol), and the methylxanthines caffeine, and theophylline [10,11]. Over 100 clinically used drugs have been described to be substrates of CYP1A2 [11]. However, many compounds are subjected to complex metabolism by several CYP enzymes so that the allover contribution of CYP1A2 is limited (~5–20%) and dominated by other pathways. Examples for drugs that are frequently and somewhat misleadingly labelled as typical CYP1A2 substrates are acetaminophen, amitriptyline, bupivacaine, carbamazepine, estradiol, fluvoxamine, haloperidol, imipramine, lidocaine, mianserin, naproxen, ondansetrone, triamterene, warfarin, and zolpidem. Although, CYP1A2 contributes to their metabolism, relevant drug-drug interactions (DDIs) cannot be expected as other metabolic pathways take over in the case of CYP1A2 inhibition. Thus, Table 3 summarizes only drugs whose systemic clearance is assumed to be >25% dependent on CYP1A2 metabolism based on the in vitro phenotyping studies and human pharmacokinetic data, as also suggested by the current Food and Drug Administration (FDA) guidance of drug–drug interactions (https://www.fda.gov/regulatory-information/search-fda-guidance-documents/vitro-drug-interaction-studies-cytochrome-p450-enzyme-and-transporter-mediated-drug-interactions). Similar to CYP3A4, CYP1A2 is a rather low affinity but high capacity metabolic enzyme. Thus, only very high concentrations of respective substrates are able to cause competitive inhibition (e.g., by extremely high doses of caffeine).

Endogenous substrates of CYP1A include arachidonic acid, bilirubin, prostaglandins, estrogens, melatonin and retinoic acid [11,126].

### 4.3. Inhibitors

Established inhibitors of CYP1A function include 7-hydroxyflavone and α-naphthoflavone that have been extensively used in vitro [11,14,18,94]. Ketoconazole, a potent inhibitor of CYP3A4 and P-glycoprotein, was also shown to inhibit CYP1A1 in a significant manner [27]. Again, we must state that there are so far insufficient data on the specific inhibitory properties of established CYP1A2 inhibitors on the function of CYP1A1. Considering the similarity in terms of sequence and function, one may hypothesize again a substantial overlap between both isoenzymes. An exception of this conclusion is furafylline, a methylxanthine, which was demonstrated to inhibit specifically CYP1A2 but not CYP1A1 [94]. Thus, it serves as an in vitro tool to distinguish between the metabolic activites of both isoenzymes in microsomal studies.

Typical inhibitors of CYP1A2 are rather small molecules, which are often heterocyclic or halogenated. Drugs resulting in potent competitive but reversible inhibition of CYP1A2 include fluoroquinolones such as ciprofloxacin and enoxacin, selective serotonin reuptake inhibiting (SSRI) antidepressants fluvoxamine and fluoxetin, the azole antimycotics ketoconazole, and clotrimazole, as well as estrogens (oral contraceptives). Some drugs (e.g., amiodarone, carbamazepine, duloxetine, isoniazid, resveratrol, and rofecoxib) were described to be mechanism-based inhibitors [11], i.e., they cause irreversible inhibition of CYP1A enzymes, which requires de novo synthesis of the respective proteins, which, in turn, results in long-lasting enzyme inhibition. Table 4 provides an overview of clinically used drugs that were identified as potent inhibitors of CYP1A. As it can be seen from the given inhibitory potential of each compound as assessed in vitro (Ki or IC_50_ values), compounds with high inhibition potency, such as ciprofloxacin (Ki, 144 nM for CYP1A2), fluxoxamine (Ki, 11-40 nM for CYP1A2), or ketoconazole (Ki, 40 nM for CYP1A1) are especially expected to cause clinically relevant drug–drug interactions [27,127,128].

## 5. Drug–Drug Interactions

Under consideration of the high number of frequently prescribed drugs that were described to be substrates (Table 3), inhibitors (Table 4), or inducers (Table 2) of human CYP1A1/A2, several unwanted drug–drug interactions can be assumed in the case of combined administration.

### 5.1. Inhibition Studies

In this regard, the most pronounced interactions have been described for the combination of CYP1A substrates with potent inhibitors including ciprofloxacin, fluvoxamine, ethinyl estradiol, and rofecoxib. Their combination with established CYP1A substrates resulted in clinically relevant interactions increasing the systemic drug exposure of caffeine, clozapine, mirtazapine, olanzapine and theophylline by 1.5 to 3-fold [106,131,142,143,144]. For agomelatine, ramelteon, tracrine, and tizanidine much more dramatic increases of serum area under the concentration-time curve (AUC) by 10–190-fold have been observed [118,119,145,146], which is expected to cause drug-related side effects and even toxicity. For example, the elevation of plasma levels of clozapine by ciprofloxacine resulted in rhabdomyolysis, delirium, and death during combination in psychotic patients [147,148].

The reasons for these dramatic interactions might be due to extensive metabolism by CYP1A enzymes and/or a high volume of distribution of the victim drug (e.g., 168 and 349 l for tizanidine and tacrine). In order to estimate the in vivo potential of a certain CYP1A inhibitor (Table 4) of an in vitro function, to cause clinically relevant interactions, focusing on the observed inhibitory potential (Ki, IC_50_ value) alone is not sufficient, but additional pharmacokinetic aspect of the perpetrator compounds must be considered as well. For sufficient inhibitory potential in vivo, a perpetrator drug needs to generate free unbound concentrations (fraction unbound, fu) around or above the observed Ki/IC_50_ value and needs to be present in the systemic circulation for several hours to cause substantial metabolic inhibition as determined by an elimination half-life of several hours. Consequently, fluvoxamine and ciprofloxacin that are characterized by rather low-to-medium protein binding (fu, 0.23 for fluvoxamine and fu, 0.8 for ciprofloxacin), but high serum levels as caused by their comparatively high administered doses (50–100 mg for fluvoxamine, 100–750 mg for ciprofloxacin) and medium to long terminal half-lives (4–7 h for ciprofloxacin, 17–22 h for fluvoxamine), cause that both drugs are strong inhibitors of CYP1A2 in vivo, and cause many clinically relevant drug–drug interactions.

This scenario is not true for other drugs mentioned in Table 4. For example, although the NSAIDs celecoxib and tolfenamic acid demonstrated a considerable inhibition of CYP1A2 in human liver microsomes (HLM) with a Ki values of 25 µM and 1.4 µM [127], they did not show clinically relevant interactions, most likely due to their high protein binding of ~98% and 99.7%, respectively. As a conclusion, drugs undergoing substantial CYP1A1/2 metabolism should be combined with caution together with the perpetrator drugs mentioned in Table 5. If possible, dose escalation combined with therapeutic drug monitoring should be used for CYP1A2 drugs with a narrow therapeutic index such as theophylline, clozapine or tizanidine. Whether the mentioned in vivo inhibitors of CYP1A2 may also cause clinically relevant interactions with CYP1A1 substrates remains uncertain and requires further studies.

### 5.2. Induction Studies

On the other side, carbamazepine, lansoprazole, omeprazole, phenobarbital, primaquine, and rosiglitazone were reported to be potent inducers of CYP1A1/1A2 by binding to AhR or CAR receptor as briefly described above [44,46,47,48,49] (Table 2), while the effects of prototypical PXR activators such as rifampicin, ritonavir and St. John’s wort are rather negligible [47,58,59,60,156]. Of these drugs, omeprazole was one of the most potent and most extensively investigated inducer in vitro and in vivo, resulting in several-fold induction of the gene expression, protein abundance and metabolic function of CYP1A1/2. However, significant effects on the pharmacokinetics and efficacy of CYP1A substrates have not been observed yet. Well-established substrates, including caffeine, phenacetin, theophylline, or propranolol did not show any changes in their pharmacokinetics in the presence of omeprazole [157,158,159,160]. Thus, one might conclude that the interaction potential of omeprazole and other proton pump inhibitors for clinically relevant DDIs might be very limited although there are also data from a case report indicating slight increase in CYP1A2 metabolism [161]. An explanation could be found in the relatively low peak concentrations of omeprazole (0.7–4.6 µM) in the systemic circulation compared to the inductive in vitro concentrations (25–50 µM) and its short half-life of 0.5–1 h (Regardh et al. 1990). In contrast to this, treatment with carbamazepine considerably induced clozapine metabolism, leading to significantly lower serum level in schizophrenic patients [149]. Carbamazepine was furthermore shown to induce hepatic caffeine metabolism as well as the systemic clearance of olanzapine and mirtazapine in a significant manner [55,162,163]. Thus, it can be stated that CYP1A2 substrates should not be combined with carbamazepine or dose-adjustment should be taken into account.

However, estimations on potential drug interactions using in vitro data on induction properties alone can be misleading. An example for that phenomenon is ritonavir, a HIV protease inhibitor. Although it showed no (or only weak) induction of CYP1A2 mRNA and activity in human hepatocytes [58,61], the pharmacokinetics of caffeine and olanzapine was significantly affected, i.e., AUC was reduced by 75% and 53% [164,165]. To overcome decreased drug efficacy due to the considerable changes in the pharmacokinetics, Jacobs et al. (2014) proposed that doubling the dose of olanzapine as a successful strategy in the case of co-medication with ritonavir [166]. The same disconnection between in vitro and in vivo effects could be observed for rifampicin, which has not been shown to be an AhR ligand and demonstrated also only a weak induction of CYP1A2 expression and metabolic function in human hepatocytes [46,47,59,61]. Accordingly, rifampicin premedication for 5–15 days reduced serum AUC of caffeine and tizanidine by 50–60% [64,164]. The reasons for this surprising finding might rely on nuclear receptor cross-talk or insensitivity of the respective in vitro on nuclear receptor activation [167]. Table 6 provides an overview about clinically relevant interactions of CYP1A substrates caused by enzyme induction.

Although it was shown in vitro experiments that CYP1A1 can be inhibited and induced by several compounds (Table 2 and Table 4), there are to the best of our knowledge no clinical drug–drug interactions that can be attributed by specific CYP1A1 inhibition or induction. However, considering the overlap in substrate recognition, inhibitors, and inducers one might speculate similar interactions as described for CYP1A2 substrates (Table 5 and Table 6). Accordingly, relevant DDIs have been estimated for CYP1A1 [179]. Nevertheless, given the low expression levels of CYP1A1 in human intestine and liver (if even), the extent of these interactions is expected to be much lower than those caused by inhibition or induction of hepatic CYP1A2.

### 5.3. Impact of Smoking and Diet

**Figure 2:**
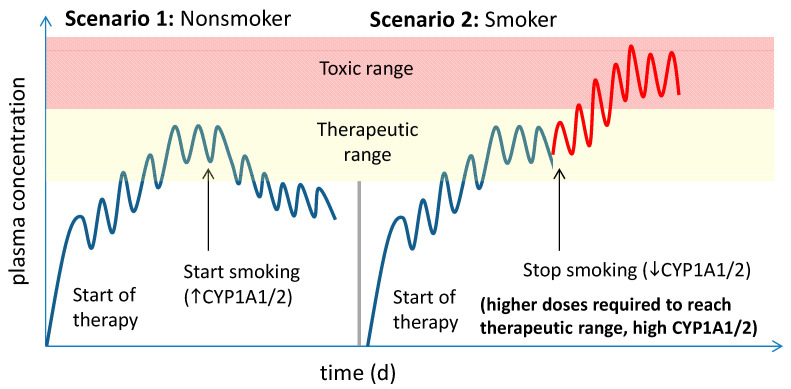
Schematic overview of potential interaction scenarios of tobacco smoking. In scenario 1, a nonsmoker reaches the steady state conditions of a certain CYP1A1/2 substrate after 5–6 half-lives using standard doses. After start smoking, CYP1A enzymes are significantly induced in intestine and liver resulting in increased drug clearance and decreasing plasma levels of the respective drug. In scenario 2, a smoker, who has already substantially higher expression and metabolic activity of CYP1A1/2, requires significantly higher doses to reach the therapeutic range. After stopping smoking, CYP1A1/2 will gradually return to the native expression levels, while the daily drug doses are not adjusted, which results in markedly increased and potentially toxic plasma concentrations.

Finally, smoking can have a profound effect on the pharmacokinetics and efficacy of several CYP1A1/2 substrates, which is comparable to potent inducing drugs, as summarized in Table 6. In all summarized examples, the systemic drug exposure of CYP1A substrates was significantly decreased in smokers compared to nonsmokers by 30–70% (Figure 2). Thus, smokers require higher doses than nonsmokers. A questionable benefit might be that smokers show also less adverse drug reactions than nonsmokers [177,180]. However, this is so far only well established in neuropsychopharmacology, i.e., treatment with antipsychotics and antidepressants. Here, individual dose adjustment is routinely performed in dependence on therapeutic drug monitoring [181].

It was shown that tobacco consumption induces CYP1A2 activity in a dose-dependent manner; smoking of daily 1–5, 6–10 and >10 cigarettes increases CYP1A2 activity 1.2-, 1.5- and 1.7-fold [182]. The maximum induction effect is already reached after smoking about 10 cigarettes daily, which abates after about three days of stopping smoking [182,183]. In particular, the latter effect may cause safety issues in the case of treatment with highly CYP1A-metabolized drugs with serious side effects, such as clozapine, olanzapine, tacrine, theophylline, or tizanidine. In this case, systemic drug exposure will substantially increase due to decreasing metabolic capacity, but unchanged high doses associated with an augmented risk for side effects, or even drug-related toxicity (Figure 2). Associated with this, cases of agranulocytosis and seizures have been reported for clozapine [181,184]. Because nicotine alone does not possess any inductive effects on CYP1A, the same risk is true in case of using e-cigarettes and other ways of nicotine substitution [185]. This should be considered by adjusting the appropriate dose, especially in case of changes in smoking habit (Figure 2).

Although a chargrilled meat diet was shown to significantly induce intestinal CYP1A1 protein as well as the metabolic activity of hepatic CYP1A2, as concluded from the caffeine breath test [33], altered pharmacokinetics of tacrine and caffeine could not be observed in a respective clinical study [186]. Some in vivo findings suggest also a potential in vivo inducing effects of broccoli [187] and another brassica vegetable, kale [188], on CYP1A2 mediated metabolism of caffeine. The brassica vegetable CYP1A2 induction is most probably mediated by 3,3′-diindolylmethane (DIM), a condensation product of indole-3-carbinol being a metabolite of the indole glucosinolate glucobrassicin. DIM has been shown to induce CYP1A2 in cultured human liver slices [189]. However, there is a lack of information about brassica vegetables interaction with clinically relevant drugs.

## 6. Summary and Conclusions

CYP1A1 and CYP1A2 are expressed in human intestine and liver. However, their inter-subject expression and function is highly variable as most likely caused by genetic, epigenetic, environmental factors (smoking, diet) and diseases. Considering the high number of drugs that have been identified as substrates, inhibitors, or inducers of CYP1A enzymes, many clinically relevant interactions have been reported and can be expected for other substrates. Thus, respective combinations should be avoided or appropriate dose adjustment is recommended in case of victim drugs with a narrow therapeutic index. In general, there is a substantial lack of data regarding CYP1A1 and its distinct role in the pharmacokinetics of drugs. However, from today’s perspective, its allover contribution to serious drug–drug interactions seems to be limited considering its low expression levels and the potential functional compensation by CYP1A2. On the other hand, CYP1A2 has to be considered as one of the big five hepatic drug metabolizing enzymes (along with CYP3A4, CYP2C9, CYP2C19, CYP2D6, CYP2E1), which is of high clinical relevance in terms of inter-subject variability of drug efficacy and safety, as well as drug–drug interactions.

## Figures and Tables

**Table 1:** Overview of available data on mRNA expression and protein abundance of cytochrome P450 (CYP) 1A1 and CYP1A2 in the human intestine and liver (+, gene/protein expression was shown; -, not investigated n.d., not detectable; PTC, proteomics; WB, Western blot). Data are ranked in chronological order (publication date).

Liver				Small Intestine				
CYP1A1		CYP1A2		CYP1A1		CYP1A2		
Gene	Protein (Method)	Gene	Protein (Method)	Gene	Protein (Method)	Gene	Protein (Method)	Reference
-	n.d. (WB)	-	+ (WB)	-	-	-	-	[20]
+	-	+	+ (WB)	-	-	-	-	[13]
-	+ (WB)	-	-	-	-	-	-	[25]
-	-	-	-	+	+ (WB)	n.d.	-	[26]
-	-	-	-	-	+ (WB)	-	-	[27]
+	-	+	-	+	-	n.d.	-	[15]
+	n.d. (WB)	+	+ (WB)	-	-	-	-	[21]
-	+ (WB)	-	+ (WB)	-	-	-	-	[14]
-	+ (WB)	-	-	-	+ (WB)	-	-	[16]
+	-	+	-	+	-	n.d.	-	[17]
-	-	+	+ (PTC)	-	-	-	-	[28]
-	+ (PTCs)	-	+ (PTC)	-	-	-	-	[23]
-	-	-	+ (PTC)	-	-	-	n.d. (PTC)	[29]
-	-	-	-	-	+ (PTC)	-	+, traces (PTC)	[30]
-	-	+	+ (PTC)	-	-	n.d.	n.d. (PTC)	[24]
-	-	-	+ (PTC)	-	-	-	-	[31]
-	-	-	-	+	-	n.d.	-	[32]
-	n.d. (PTC)		+ (PTC)	-	n.d. (PTC)	-	n.d. (PTC)	[22]
-	+ (PTC)	-	+ (PTC)	-	-	-	-	[18]

**Table 2:** Impact of clinically relevant drugs, smoking, and diet on the induction of CYP1A1/1A2 mRNA, protein, and activity.

Drug	Object of Investigation	Induction Effect	Reference
Albendazole (5–30 µM)	HepG2 cells	↑CYP1A1 (32-fold) and CYP1A2 mRNA (5.6-fold); ↑EROD-activity (4-fold)	[49,50]
Carbamazepine (7–183 µM)	HepaRG cells	↑CYP1A2 mRNA (10-fold)	[52]
Carbamazepine	Pediatric patients	↑hepatic CYP1A2 activity (CBT, 2.2-fold)	[55]
Lansoprazole (50 µM)	Human hepatocytes	↑CYP1A2 mRNA (26-fold); ↑CYP1A2 protein (32-fold); ↑EROD activity (32-fold)	[48]
Omeprazole (0.03–3 µM)	Human hepatocytes	↑CYP1A1 mRNA (37-fold)	[47]
Omeprazole (50 µM)	Human hepatocytes	↑CYP1A2 mRNA (12-fold); ↑CYP1A2 protein (4.6-fold); ↑EROD activity (39-fold)	[48]
Omeprazole (25 µM)	HepG2 cells	↑CYP1A1 and CYP1A2 mRNA	[49]
Omeprazole (treatment)	Human duodenal biopsies	↑CYP1A1 protein; ↑EROD activity (2.2-fold)	[56]
Omeprazole (20 mg SID, 4 d)	Human liver biopsies	↑CYP1A2 protein (3.4-fold); ↑EROD activity (6-fold)	[57]
Phenobarbital (100–250 µM)	Human hepatocytes	↑EROD activity (1.9-fold)	[46]
Phenobarbital (1 mM)	Human hepatocytes	↑CYP1A2 mRNA (1.5-fold); ↑CYP1A2 protein (1.8-fold); ↑POD activity (3.1-fold)	[58]
Primaquine (10–30 µM)	HepG2 cells	↑CYP1A1 (~7-fold) and CYP1A2 (~3-fold) mRNA; ↑EROD-activity (7.5-fold)	[49,50]
Quinine (30 µM)	HepG2 cells	↑CYP1A1 (~9-fold) and CYP1A2 mRNA (2.4-fold); ↑EROD-activity (5.5-fold)	[50]
Rifampicin (10/33 µM)	Human hepatocytes	↑CYP1A1 (2.2-fold) and CYP1A2 mRNA (2.2-fold)	[47,59,60,61]
Rifampicin (20/50 µM)	Human hepatocytes	↑EROD-activity (2.3-fold)	[46]
Ritonavir (0.1–5 µM)	Human hepatocytes	±CYP1A2 mRNA (0.8-fold); ±CYP1A2 protein (1.0-fold); ↑POD activity (1.6-fold)	[58]
Ritonavir (1–25 µM)	Human hepatocytes	↑CYP1A2 mRNA (4-fold); ↑POD activity (2-fold)	[61]
Rosiglitazone (10 µM)	Human hepatocytes	↑CYP1A2 mRNA (11-fold); ↑CYP1A2 protein (7-fold); ↑EROD activity (37-fold)	[48]
Smoking	Human liver biopsies	↑EROD activity (3.3-fold)	[62]
Smoking (3–30/d), 7d	Human duodenal biopsies	↑EROD activity (4.2-fold)	[56]
Chargrilled meat diet (7 d)	Human duodenal biopsies	↑CYP1A1 protein, ↑hepatic CYP1A2 activity (CBT, 1.9-fold)	[33]

↑, increase; ±, unchanged; CBT, caffeine breath test; EROD, 7-ethoxyresorufin O-deethylase; POD, phenacetin O-deethylation.

**Table 3:** Overview for clinically relevant drugs undergoing significant CYP1A2-mediated metabolism (≥25%).

Substrate	Drug Class	Metabolic Reaction	Contribution of CYP1A2 (Other CYPs)	Reference
Aminopyrine	Analgesic drug	N-demethylation	40–50% (CYP2C8/2C19)	[98]
Agomelatine	melatonin receptor agonist (antidepressant)	hydroxylation and demethylation	90% (10% CYP2C9)	[99]
Caffeine	CNS stimulant	N-demethylation	>95%	[94,95]
Clozapine	Atypical antipsychotic drug	N-demethylation and N-oxidation	40–55% (CYP3A4/2C19)	[100]
Dacarbazine	Anticancer drug	N-demethylation	20–40% (CYP1A1/2E1)	[101]
Duloxetine	Antidepressant	4-, 5- and 6-hydroxylation major extent substrate	30–40% (CYP2D6/2C9)	[102]
Flutamide	Non-steroidal antiandrogen	2-Hydroxylation	~25% (CYP3A4/2C19)	[103]
Leflunomide	Disease-modifying anti-inflammatory drug	N-O bond cleavage	40–55%	[104]
Melatonin	Pineal hormone	6-hydroxylation and O-demethylation	40–60% (CYP1A1/1B1)	[105]
Mirtazapine	Antidepressant	8-hydroxylation and N-demethylation	30–50% (CYP3A4/2D6)	[106]
Nabumetone	NSAID	aliphatic hydroxylation	30–40% (CYP2C9)	[107]
Olanzapine	Atypical antipsychotic drug	N-demethylation and 7-hydroxylation	30–40% (CYP2D6)	[108]
Phenacetin	Analgesic drug	O-deethylation and C-hydroxylation	86%	[94]
Promazine	Antipsychotic drug	N-demethylation and 5-sulfoxidation	30–45% (CYP2C19/3A4)	[109]
Propranolol	β-Blocker	N-deisopropylation, and 4- and 5-hydroxylation	30–50% (CYP2D6)	[110]
Ramelteon	Melatonin receptor agonist (hypnotic)	Aliphatic hydroxylation	~50% (CYP2C19/3A4)	[111]
Rasagiline	Antiparkinson drug	N-dealkylation and hydroxylation	>50%	[112]
Riluzole	Antiglutamate agent (treatment of ALS)	N-hydroxylation	~80%	[113]
Ropinirole	Antiparkinson drug	N-depropylation and hydroxylation (major)	30–45%	[114]
Ropivacaine	Local anesthetic drug	3-, 4-hydroxylation	50–65% (CYP3A4)	[115]
Tacrine	cholinesterase inhibitor (Alzheimer’s disease)	1-, 2-, 4- and 7-Hydroxylation	50–65%	[116]
Theophylline	Bronchodilator (Asthma/COPD)	N-demethylation	90–95%	[117]
Tizanidine	Muscle relaxant	Hydroxylation	80–95%	[118,119]
Verpamil	Calcium channel blocker	N-demethylation and N-dealkylation	20–30% (CYP2C8/3A4)	[120]
Zolmitriptan	Selective 5-HT_1B/1D_ (treatment of migraine)	N-demethylation and O-demethlyation	30–40%	[121]

5-HT, 5-hydroxy tryptamine; ALS, Amyotrophic lateral sclerosis; CNS, central nervous system; COPD, Chronic obstructive pulmonary disease; NSAID, non-steroidal anti-inflammatory drug.

**Table 4:** Overview of clinically relevant drugs with inhibitory properties on CYP1A1/1A2.

Drug	Drug Class	In Vitro System	Inhibitory Effect (Isoenzyme)	Reference
Alosetron ^1^	5HT_3_-receptor antagonist (irritable bowel syndrome)	HLM	IC_50_ = 2 µM (CYP1A2)	[129]
Amiodarone ^1^	Antiarrhythmic drug	HLM	IC_50_ = 86 µM	[130]
Artemesinin ^1^	Antimalaria drug	HLM	Ki = 0.43 μM (CYP1A2)	[131]
Carbamazepine ^2^	Anticonvulsant	HLM	n.d. (CYP1A2)	[132]
Celecoxib ^1^	COX-2 inhibitor	HLM	Ki = 25.4 µM (CYP1A2)	[127]
Ciprofloxacin ^1^	Antibiotic (fluoroquinolone)	HLM HLM	70.4% (CYP1A2) Ki = 144 nM (CYP1A2)	[133] [127]
Cimetidine ^1^	H_2_-receptor antagonist	HLM	Ki = 200 µM (CYP1A2)	[134]
Clotrimazole ^1^	Antifungal agent	human lymphoblast cells	Ki = 7.9 µM (CYP1A2)	[135]
Desogestrel ^1^	Hormone (oral contraceptive)	HLM	Ki = 39.4 µM (CYP1A2)	[127]
Duloxetine ^2^	Antidepressant (SSRI)	HLM	n.d. (CYP1A2)	[136]
Enoxacin ^1^	Antibiotic (fluoroquinolone)	HLM	74.9% (CYP1A2)	[133]
Ethinyl estradiol ^1^	Hormone (oral contraceptive)	HLM	Ki = 10.6 µM (CYP1A2)	[127]
Fluoxetine ^1^	Antidepressant (SSRI)	HLM	Ki = 4.4 µM (CYP1A2)	[137]
Fluvoxamine ^1^	Antidepressant (SSRI)	HLM	Ki = 33 µM (CYP1A1) Ki = 40 nM (CYP1A2) Ki = 11 nM (CYP1A2)	[128] [128] [127]
Isoniazid ^2^	Antibiotic	HLM	Ki = 285 µM (CYP1A2)	[138]
Ketoconazole ^1^	Antifungal agent	HIM HLM	Ki = 40 nM (CYP1A1) IC_50_ = 0.33 µM CYP1A2)	[27] [139]
Miconazole ^1^	Antifungal agent	human lymphoblast cells	Ki = 3.2 µM (CYP1A2)	[135]
Nifedipine ^1^	Calcium channel blocker	HLM	n.d. (CYP1A1) n.d. (CYP1A2)	[94]
Norfloxacin ^1^	Antibiotic (fluoroquinolone)	HLM	55.7% (CYP1A2)	[133]
Paroxetine ^1^	Antidepressant (SSRI)	HLM	Ki = 5.5 µM (CYP1A2)	[137]
Propafenone ^1^	Antiarrhythmic drug	HLM	IC_50_ = 29 µM	[130]
Propranolol	Beta blocker	HLM	n.d. (CYP1A1) n.d. (CYP1A2)	[94]
Resveratrol ^2^	Natural compound	HLM	IC_50_ = 23 µM (CYP1A1) Ki = 2.2 µM (CYP1A2)	[140] [141]
Riluzole ^1^	Amyotrophic lateral sclerosis drug	HLM	Ki = 12.1 µM (CYP1A2)	[113]
Rofecoxib ^2^	COX-2 inhibitor	HLM	Ki = 6.2 µM (CYP1A2)	[127]
Sertraline ^1^	Antidepressant (SSRI)	HLM	Ki = 8.8 µM (CYP1A2)	[137]
Sulconazole	Antifungal agent	human lymphoblast cells	Ki = 0.4 µM (CYP1A2)	[135]
Thiabendazol ^2^	Antifungal/antiparasitic agent	HLM	Ki = 1.54 μM (CYP1A2)	[131]
Tioconazole ^1^	Antifungal agent	human lymphoblast cells	Ki = 0.4 µM (CYP1A2)	[135]
Tolfenamic acid ^1^	NSAID	HLM	Ki= 1.4 µM (CYP1A2)	[127]

^1^ Competitive (reversible) inhibitor; ^2^ mechanism-based (irreversible) inhibitor; 5-HT, 5-hydroxy tryptamine; COX, cyclooxygenase; HLM, human liver microsomes; IC_50_, half maximal inhibitory concentration; Ki, inhibition constant; NSAID, nonsteroidal anti-inflammatory drug; SSRI, selective serotonin reuptake inhibitor.

**Table 5:** Overview of clinically relevant interaction as caused by inhibition of CYP1A1/1A2 enzymes.

Substrate (Victim Drug)	Perpetrator (Inhibitor)	PK Change	Reference
Agomelatine	Fluvoxamine	AUC ↑ 60-fold	Product information
Caffeine (137 mg, SD)	Thiabendazol (500 mg, SD)	AUC ↑ 1.6-fold t_1/2_ ↑ 2.4-fold	[131]
Clozapine (50–700 mg)	Fluvoxamine (50–100 mg, SID, MD)	C_SS_ ↑ 5-10-fold	[149]
Clozapine (2.5–3.0 mg/kg)	Fluvoxamine (50 mg, SID, MD)	C_SS_ ↑ 3-fold	[150]
Clozapine (200–350 mg)	Fluvoxamine (50 mg, SID, MD)	C_SS_ ↑ 2.2-fold	[142]
Clozapine (150–400 mg)	Ciprofloxacin (250 mg BID, 7 d)	C_SS_ ↑ 1.3-fold	[151]
Duloxetine (60 mg, SD)	Fluvoxamine (100 mg SID, 16 d)	AUC ↑ 5.6-fold C_MAX_ ↑ 2.4-fold	[102]
Melatonin (5 mg, SD)	Fluvoxamine (50 mg, SD)	AUC ↑ 17-fold C_MAX_ ↑ 12-fold	[105]
Mirtazapin (15–30 mg)	Fluvoxamine (50–100 mg, SID, MD)	C_SS_ ↑ 1.3-fold	[106]
Olanzapine (10 mg, SD)	Fluvoxamine (100 mg, SID, 14 d)	AUC ↑ 1.5-fold C_MAX_ ↑ 1.6-fold	[143]
Propranolol (160 mg, SID)	Fluvoxamine (100 mg)	C_MAX_ ↑ 5-fold	[152]
Ramelteon (16 mg, SD)	Fluvoxamine (100 mg BID, 3 d)	AUC ↑ 190-fold C_MAX_ ↑ 70-fold	Product information [111]
Ropivacaine (0.6 mg/kg, iv)	Ciprofloxacin (500 mg BID, 2.5 d)	CL ↓ 31%	[153]
Tacrine (40 mg, SD)	Fluvoxamine (100 mg SID, 6 d)	AUC ↑ 8.3-fold C_MAX_ ↑ 5.6-fold	[145]
Theophylline (250 mg, SD)	Fluvoxamine (75 mg, SD)	AUC ↑ 2.4-fold t_1/2_ ↑ 2.5-fold	[144]
Theophylline (3.4 mg/kg, SD)	Ciprofloxacin (500 mg BID, 3 d)	CL ↓ 19% t_1/2_ ↑ 26%	[154]
Tizanidine (4 mg, SD)	Rofecoxib (25 mg SID, 4d)	AUC ↑ 13.6-fold C_MAX_ ↑ 6.1-fold	[146]
Tizanidine (4 mg, SD)	Ciprofloxacin (500 mg BID, 3 d)	AUC ↑ 10-fold C_MAX_ ↑ 7-fold	[118]
Tizanidine (4 mg, SD)	Fluvoxamine (100 mg SID, 4d)	AUC ↑ 33-fold C_MAX_ ↑ 12-fold	[119]
Tizanidine (4 mg, SD)	Ethinyl estradiol 20–30 µg, gestodene 75 µg	AUC ↑ 3.9-fold C_MAX_ ↑ 3-fold	[155]
Ropivacaine (0.6 mg/kg, iv)	Ciprofloxacin (500 mg BID, 2.5 d)	CL ↓ 31%	[153]

↑, increase; ↓, decrease; AUC, area under the concentration-time curve; BID, twice daily; CL, clearance; Cmax, maximum serum concentration; Css, trough serum concentrations at steady-state; d, days; MD, multiple doses; PK, pharmacokinetic; SID, once daily; SD, single dose; t1/2, elimination half-life.

**Table 6:** Overview of clinically relevant interaction as caused by induction of CYP1A1/1A2 enzymes.

Substrate (Victim Drug)	Perpetrator	PK Change	Reference
Antipyrine (20 mg/kg, SD)	Smoking	CL ↑ 46%	[62]
Caffeine (100 mg, SD)	Carbamazepine (400 mg, SID, 14 d)	CL ↑ 27–47%	[163]
Caffeine (2 mg/mg, SD)	Lopinavir (400 mg)/Ritonavir (100 mg), BID, 14 d	MR ↑ 43%	[168]
Caffeine (200 mg, SD)	Rifampicin (400 mg BID, 14 d)	AUC ↓ 60% CL ↑ 214%	[164]
Caffeine (200 mg, SD)	Ritonavir (400 mg BID, 14 d)	AUC ↓ 75% CL ↑ 290%	[164]
Clozapine (150–900 mg)	Smoking (7–>20/d)	C_SS_ ↓ 50% C_SS_ ↓ 40%	[169] [170]
Clozapine (325 mg)	Omeprazole (40–60 mg, MD)	C_SS_ ↓ 42–45%	[161]
Clozapine	Carbamazepine	C_SS_ ↓ 50%	[149]
Duloxetine (86–102 mg, MD)	Smoking	C_SS_ ↓ 53%	[171]
Estradiol (1–2 mg)	Smoking	C_SS_ ↓ 43%	[172]
Mirtazapine (15–30 mg, SID, 7 d)	Carbamazepine (200-400 mg, BID, 21 d)	AUC ↓ 63% C_max_ ↓ 44%	[173]
Mirtazapine (30 mg, SID, 28 d)	Smoking	C_SS_ ↓ 41%	[174]
Olanzapine	Carbamazepine	CL ↑ 46% t_1/2_ ↓ 20%	[162]
Olanzapine (10 mg, SD)	Rifampicin, 600 mg, SID, 7 d	AUC ↓ 48% C_max_ ↓ 11%	[175]
Olanzapine	Ritonavir (300–500 mg BID, 3–5 d)	AUC ↓ 53% C_MAX_ ↓ 40%	[165]
Olanzapine	Smoking (light, 1–4/d) Smoking (medium, >5) Smoking (heavy, 7–>20)	AUC ↓ 45% AUC ↓ 68% C_SS_ ↓ 67%	[176] [176] [169]
Theophylline	Smoking	CL ↑ 58–100% t_1/2_ ↓ 63%	[177]
Tizanidine	Rifampicin, 500 mg, SID, 5 d	AUC ↓ 53%	[64]
Tizanidine	Smoking	AUC ↓ 33%	[68]
Verapamil	Smoking	AUC ↓ 20%	[178]

↑, increase; ↓, decrease; AUC, area under the concentration-time curve; BID, twice daily; CL, clearance; C_max_, maximum serum concentration; Css, trough serum concentrations at steady-state; d, days; MD, multiple doses; MR, metabolic ratio; PK, pharmacokinetic; SID, once daily; SD, single dose; t_1/2_, elimination half-life.
